# Irreversible antidepressant-induced sexual dysfunction: what do we know so far?

**DOI:** 10.1192/j.eurpsy.2025.2296

**Published:** 2025-08-26

**Authors:** A. Cardoso, M. Mota

**Affiliations:** 1 Psychiatry Department, Unidade Local de Saúde de São João, Porto, Portugal

## Abstract

**Introduction:**

Serotonergic antidepressants, particularly Selective Serotonin Reuptake Inhibitors (SSRIs), have been known to cause relevant sexual adverse effects, which were originally thought to disappear after treatment course. In later years, an emergent condition concerning the persistence of sexual impairment following SSRI discontinuation has gathered increasing social and scientific interest. Post-SSRI sexual dysfunction (PSSD) is still underrecognized and understudied.

**Objectives:**

Our aim was to gather available evidence regarding the pathophysiological mechanisms and contributing factors to PSSD, as well as current management options.

**Methods:**

A literature review was conducted using PubMed, through research of the following MESH terms: “Selective Serotonin Reuptake Inhibitors”, “Antidepressive Agents”, “Sexual Dysfunction, Physiological” and “Sexual Dysfunctions, Psychological”. Only papers published in English were included.

**Results:**

PSSD has been described as an iatrogenic condition whose symptoms could persist indefinitely. Despite clinical heterogeneity and lack of robust literature surrounding PSSD, there is growing evidence of this concern. In order to diagnose PSSD, sexual dysfunction due to reemergence of depressive illness must be ruled out. Accurate incidence and prevalence rates of this condition are unknown, though it appears to be a rare phenomenon. Several potential etiological explanations for PSSD have been proposed. Some have highlighted a dysregulation in serotonergic activity induced by SSRIs, while others have tested the role of neuroregulatory agents and bioelectric circuits. Another theory involves a possible link between PSSD and other post-discontinuation syndromes. In contrast, some authors have theorized that sexual impairment induced by a long course of SSRIs could induce a negative conditioning effect towards sexual activity in some patients. Despite abovementioned hypotheses, sexual dysfunction is usually complex in nature and thus multifactorial. There is no established treatment to PSSD. Pharmacological and psychotherapeutic approaches have been tested and proposed to ameliorate symptoms of PSSD, but further investigation is warranted.

**Conclusions:**

PSSD is a heterogenous and idiosyncratic syndrome that needs further characterization. It remains unclear why only certain individuals develop PSSD and treatment options are limited. Current evidence is still incipient and insufficient to justify adjusting current SSRI prescription patterns. However, clinicians must be aware of this condition and should monitor and address these issues with patients. Future research should provide further insights into underlying pathophysiological mechanisms, which in turn could help acknowledge possible preventive and therapeutic interventions.

**Disclosure of Interest:**

None Declared

